# A cost-utility analysis of pregabalin versus venlafaxine XR in the treatment of generalized anxiety disorder in Portugal

**DOI:** 10.1186/1478-7547-11-8

**Published:** 2013-04-12

**Authors:** Luís Silva Miguel, Nuno Silva Miguel, Mónica Inês

**Affiliations:** 1 Research Centre on the Portuguese Economy (CISEP), Instituto Superior de Economia e Gestão, Technical University of Lisbon, Lisbon, Portugal; 2 Ares do Pinhal, Mação, Portugal; 3 Pfizer, Porto Salvo, Portugal

## Abstract

**Background:**

Generalized anxiety disorder is characterized by excessive anxiety and worry about several events and activities. The estimated 1-year prevalence for adults is around 2% and the lifetime prevalence could reach more than 6%. The disease is associated with reduced quality of life, being comparable to that of major depressive disorder and to chronic illnesses such as diabetes and arthritis, and high consumption of health care resources.

**Methods:**

A previously published patient-level simulation cost-utility model was adapted to the Portuguese context in order to evaluate clinical and economic consequences of using pregabalin in place of venlafaxine XR in the treatment of generalized anxiety disorder. The model predicts the evolution of 1,000 patients with generalized anxiety disorder, simulating their pathway in weekly cycles over one year treatment. This is done by setting a pre-treatment Hamilton Anxiety Scale score and projecting the weekly impact of the pharmacotherapy on this score. The model uses clinical data from an 8-week flexible dose direct comparison clinical trial between the two drugs; utility values based on a Spanish study; and Portuguese economic data, being the resource consumption obtained via an expert panel.

**Results:**

Pregabalin patients benefited from 0.738 quality adjusted life years while those on venlafaxine XR achieved 0.712. Moreover, the number of weeks with no or minimal anxiety symptoms was estimated to be 12.9 for pregabalin and only 3.8 for venlafaxine XR. Those clinical gains were achieved at the expense of an extra 715€ per patient, implying an incremental cost per quality adjusted life year of 27,199€ and an incremental cost per week with no or minimal symptoms of 79€. Sensitivity analysis shows that results are robust to main assumptions.

**Conclusions:**

Assuming a threshold of 30,000€ per quality adjusted life year, pregabalin is cost-effective in comparison with venlafaxine XR in the treatment of generalized anxiety disorder in Portugal.

## Background

Generalized anxiety disorder (GAD) is mainly characterized by at least six months of excessive anxiety and worry, or apprehensive expectation, about a number of events and activities, with these feelings being difficult to control and occurring more days than not. It is also characterized by symptoms including restlessness, fatigue, impaired concentration, irritability, muscle tension and sleep disturbances [1].

A review on the available epidemiological data about GAD in Europe, that included 15 studies reporting data for 15 countries, concluded that the estimated 1-year prevalence in the adult population is around 2%, being the median of included studies 1.7% [2]. The estimates of lifetime prevalence are less consistent, varying from 0.1% to 6.4%. This review also suggests a higher risk amongst women (2–3 fold versus men). GAD is one of the most frequent mental disorders in primary care, despite the fact of its recognition in this setting being relatively low, and leads to a high use of healthcare resources [2].

The disease has also been associated with reduced quality of life [3,4], being comparable to major depressive disorder and to other chronic illnesses, such as diabetes and arthritis [5]. In terms of occupational and social functioning, it is important to note, for example, that in a German study almost a third of GAD patients reduced their annual productivity by more than 10%, while only 8% of those with major depression did so [6].

Several drugs with different pharmacological properties have shown their efficacy in the treatment of GAD in placebo controlled trials. Traditionally, benzodiazepine drugs were used in this context but their potential to cause dependence led to restrictions in the duration of use, being only recommended for short term use. Other pharmacotherapies that have been used to treat GAD include selective serotonin reuptake inhibitors (SSRIs), such as paroxetine; serotonin noradrenaline reuptake inhibitors (SNRIs), such as extended-release (XR) venlafaxine; and an anticonvulsant agent, pregabalin [7].

According to the latest National Clinical Guideline issued by the National Institute of Health and Clinical Excellence (NICE), the most cost-effective option in the English and Welsh settings is sertraline, and only if this proves ineffective should another SSRI or venlafaxine be provided. Only if the patient does not tolerate SSRIs or SNRIs does NICE recommend the prescription of pregabalin [8].

Given the recommendations of NICE have a worldwide impact, it is important to confirm if their findings are transferable to other settings and reinforced by other pharmacoeconomic models. Therefore, in this study we report the results of a pharmacoeconomic model of GAD treatment that has been previously used in the Spanish context [9]. The objective of the present study is to evaluate the cost-utility of pregabalin versus venlafaxine XR in the Portuguese context.

## Methods

### The pharmacoeconomic model

A patient-level simulation model developed by Policy Analysis Inc. [9] using Microsoft Excel © was adapted to the Portuguese context in order to evaluate clinical and economic consequences of using pregabalin instead of venlafaxine XR in the management of patients with GAD. A patient-level simulation is a type of pharmacoeconomic model that allows simulating the entire path of several patients with a set of unique characteristics, through the use of individual data. It contrasts with a cohort simulation, in which the evolution of a group of patients is simulated assuming that all patients behave as the “average” individual.

In the present model, patients are categorized according to the Hamilton Anxiety Scale (HAM-A), an instrument that allows clinicians to rate symptoms and, therefore, the severity of the underlying disease [10]. The rating is done scoring on a 5 point Likert scale – from 0 (not present) to 4 (very severe) – each of the following fourteen items: anxious mood, tension, fears, insomnia, intellectual difficulties, depressed mood, somatic muscular and sensory complaints, cardiovascular, respiratory, gastrointestinal, genitourinary and autonomic symptoms, and patient’s behavior during the interview. Scores range from 0 to 56.

For modelling purposes, generalized anxiety disorder was grouped in four categories: “no or minimal anxiety” (HAM-A score ≤ 9), “mild anxiety” (10–15), “moderate anxiety” (16–24), and “severe anxiety” (≥ 25). Following the characteristics of patients included in most clinical trials, the model considers that before implementation of any pharmacotherapeutic option all patients have either moderate or severe anxiety. Clinical gains are achieved whenever disease management allows subjects to be classified in less severe categories, following the rationale of valuing time without or with lighter symptoms that was already applied in economic evaluations of medical conditions as pain, depression and epilepsy [11-15].

**Figure 1:**
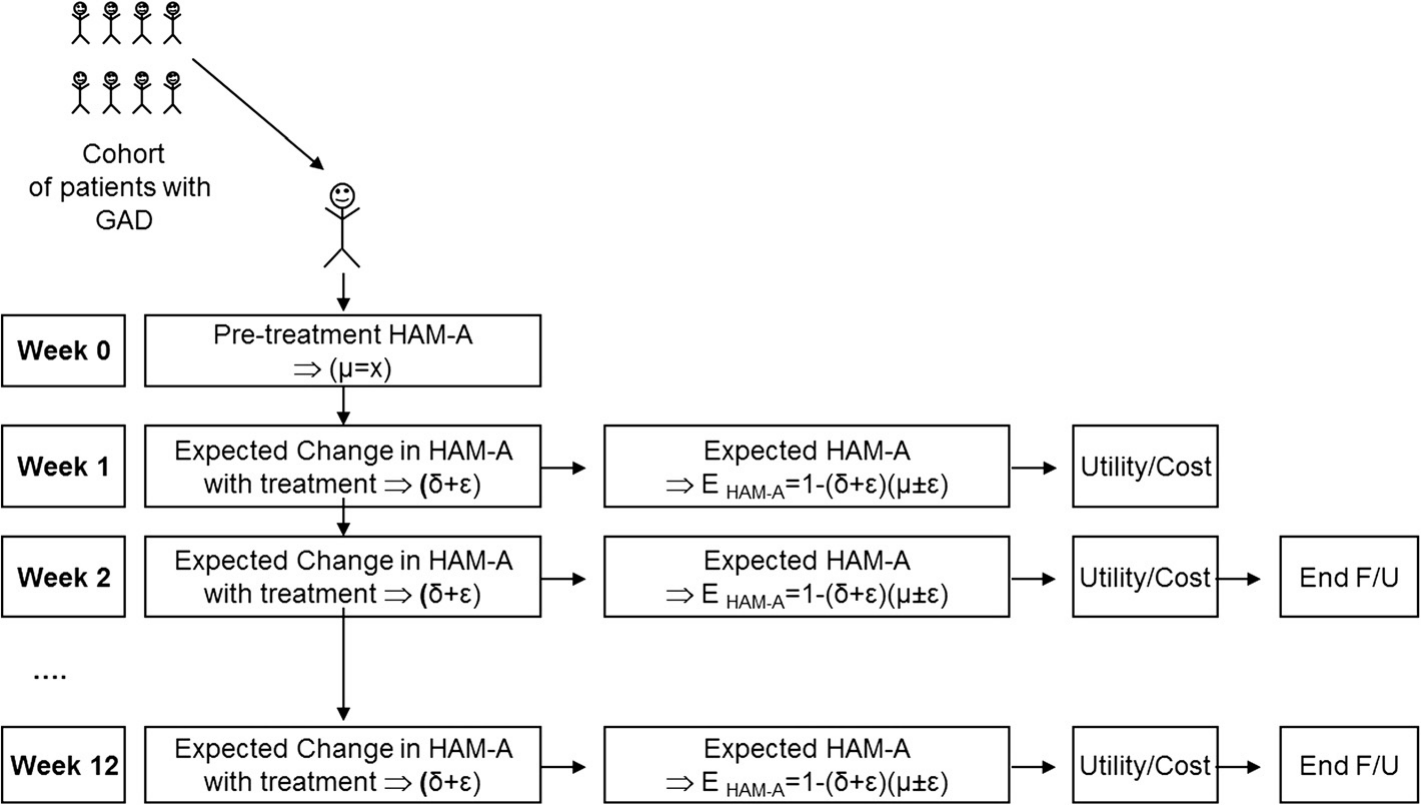
Patient level simulation model.

The model predicts the evolution of a hypothetical cohort of 1,000 patients with GAD, simulating their pathway in weekly cycles over a time-frame of one year. This is done by sampling (with replacement) from the pre-treatment HAM-A scores and projecting the weekly impact of the pharmacotherapy on this score up to one year, using expected change from baseline (also through sampling with replacement). The score change replicates clinical findings, being possible to assume different proportional gains in each week (e.g. a lower impact in the first weeks after therapy implementation) as well as different gains per patient, as not all patients benefit from treatment to the same extent. The multiplication of the initial score by the percentage change from baseline allows calculation of the new HAM-A score, which may or may not belong to the same GAD severity category, as defined above. Therefore, as each patient moves through the *continuum* of HAM-A scores, the respective quality of life and healthcare resource consumption may or may not differ according to the initial HAM-A score and the weekly change. It should be stressed that this kind of modelization is only possible when implementing patient-level simulations. It is also important to note that the model allows patients to stop treatment (either due to adverse events or lack of efficacy) and to switch to another drug. The model is illustrated on Figure 1.

Despite the fact that the main clinical measure is quality adjusted life years (QALY), the model allows to calculate other measures of outcomes, as the mean HAM-A and the number of weeks with no or minimal symptoms. On the economic side, the model estimates total costs for the time horizon specified. Therefore it is possible to estimate the cost per clinical gain, namely the cost per quality adjusted life year.

Finally, concerning the uncertainty inherent to all economic evaluations, besides the usual one-way sensitivity analysis that in this study evaluates the impact of different time horizons, assumptions on discontinuation and switch, utility values, and costs, it is possible to run a probabilistic sensitivity analysis on the weekly mean percentage change from baseline, allowing to estimate a cost-utility acceptability curve. For this analysis, the model was run for 100 samples of 1,000 patients each, assuming a left-truncated normal distribution, as percentage mean reduction could not be higher than 100%.

### Clinical data

As the objective of this analysis is to compare the utilization of pregabalin and venlafaxine XR in the management of patients with generalized anxiety disorder, clinical data was obtained on a single direct comparison clinical trial between these two drugs. The PEACE study [16] is an 8-week, multicenter, randomized and double blind clinical trial of pregabalin (300-600 mg/day), venlafaxine XR (75-225 mg/day), and placebo. The flexible dose regimen allows to simulate conditions of typical clinical practice, increasing the external validity of the findings.

The intention-to-treat sample, considered for efficacy and safety analysis, was composed by 374 patients, from whom 121 were assigned to pregabalin, 125 to venlafaxine XR and 128 to placebo, without significant demographic or clinical differences at baseline. The distribution of pre-treatment HAM-A scores is shown on Table 1 (data on file). It can be seen that almost 75% of individuals had severe GAD. Results show that pregabalin enabled a decrease of 14.5 points on the HAM-A score since baseline, that compares with 12.0 and 11.7 for venlafaxine XR and placebo, respectively. The mean changes from baseline for pregabalin and venlafaxine XR non-quitters patients are available on Table 2 (data on file). For modelling purposes, as the trial only lasts for 8 weeks, it was assumed that the score achieved at week 8 was maintained through the rest of the year.

**Table 1:** Pre-treatment HAM-A Score

**Anxiety state**	**HAM-A score**	**Proportion of patients**
Moderate	16-19	0.8%
	20-24	24.7%
Severe	25-34	66.4%
	35-44	7.8%
	45-56	0.3%

Source: data on file.

**Table 2:** Mean changes from baseline (%)

**Week**	**Pregabalin**			**Venlafaxine XR**		
	**Mean**	**SD**	**SE**	**Mean**	**SD**	**SE**
1	−27.2	20.6	2.22	−18.7	20.2	2.08
2	−39.8	22.1	2.38	−34.7	24.3	2.51
3	−45.7	24.1	2.60	−43.0	24.9	2.56
4	−51.4	24.1	2.60	−48.3	25.9	2.67
5	−51.4	24.1	2.60	−48.3	25.9	2.67
6	−57.4	26.5	2.86	−51.5	26.0	2.69
7	−57.4	26.5	2.86	−51.5	26.0	2.69
8	−61.4	26.0	2.81	−52.6	26.0	2.68

Source: data on file.

Given that one of the important aspects of this genre of therapy is the speed of onset, this aspect was also evaluated in the trial. At day 4, the difference between pregabalin and the other options was significant, with 36.3% achieving a reduction higher than 20% in the HAM-A score, while only 18.3% of those taking venlafaxine XR and 20.3% of the ones on placebo achieved such a reduction. Mean (±SD) daily dose was 348 mg (±85) for pregabalin and 102 mg (±33) for venlafaxine XR. About 30% of patients discontinued treatment on each arm, with more quitters for venlafaxine XR, mainly due to a worst adverse events profile. On the pregabalin arm, 3.3% discontinued due to lack of efficacy, 12.4% due to adverse events, and 11.6% for other reasons. On the venlafaxine XR and placebo arms, the figures were 3.2%, 17.6% and 12.0%; and 9.4%, 5.5% and 12.5%, respectively.

### Utility scores

Health-related utility values were based on the EQ-5D application on a cross-sectional study of 456 Spanish individuals with GAD diagnosis, randomly selected from 134 primary health centers (Table 3) [17]. EQ-5D values were mapped to 4 levels of disease severity as measured by the HAM-A. Bearing in mind the geographic proximity and cultural similitude between Portugal and Spain, the use of these figures seems reasonable.

**Table 3:** Health-state utility values by HAM-A score

**HAM-A Score**	**EQ-5D Utility**
≤ 9	0.84
10 - 15	0.71
16 – 24	0.68
≥ 25	0.53

Source: [17].

### Economic data

Resource consumption estimates were based on an expert panel of five Portuguese psychiatrists with large clinical experience in managing patients with GAD. The consensus achieved is shown on Table 4, while unit costs for each resource are displayed on Table 5. This study was undertaken from the payers perspective in Portugal, whether they were public or private, implying that the full cost of each resource should be included. Drug costs were calculated using official prices [18] weighted by each presentation market share [19]. Exams, analyses, inpatient stays and emergency visits were also valued according to official sources [20,21]. Doctor visits costs were calculated as a weighted average of public [21] and private prices [22] for consultations, according to the weights derived from the most recent National Inquiry of Health, whose data is from 2005/06 [23]. Thus, the weekly costs per patient used in the model were 12€ for none or minimal, 16€ for mild, 27€ for moderate, and 40€ for severe GAD. It should be noted that these values are an average of both alternatives and are therefore conservative, as the panel indicated that those patients on venlafaxine XR tend to consume more expensive concomitant drugs than those taking pregabalin. Moreover, they do not include the costs of pregabalin and venlafaxine XR: 2.80€ and 0.72€ per day, respectively (assuming the mean doses estimated in the clinical trial and a weighted average of generic and brand prices of venlafaxine XR). Productivity costs were not included, meaning that it was assumed that there are no productivity gains due to the decrease in GAD symptoms. Obviously, this is a conservative assumption in the sense that it leads to an underestimation of the benefits associated to the most efficacious option.

**Table 4:** Resource consumption per patient, during each 4 weeks, by HAM-A score

	**HAM-A Score**			
	**≤ 9**	**10 - 15**	**16 - 24**	**≥ 25**
Number of doctor visits				
Number of general practitioner visits	0.6	0.8	1.2	1.6
Number of psychiatrist visits	0.08	0.1	0.2	0.4
Number of other specialist visits	0.15	0.2	0.3	0.4
Number of psychologist visits	0	0	0.1	0.1
Number of emergency attendances	0.05	0.1	0.15	0.25
Number of exams and analyses				
Number of electrocardiograms	0.3	0.4	0.6	0.8
Number of blood analyses	0.3	0.4	0.6	0.8
Number of thyroid functions	0	0	0.05	0.05
Number of inpatient stays	0	0	0	0.004
Concomitant drugs for both alternatives				
Proportion taking antipsychotics	0%	0%	5%	10%
Most prescribed	Quetiapine			
Mean daily dose	-	-	100 mg qd	100 mg qd
Proportion taking hypnotics	0%	0%	10%	20%
Most prescribed	Zolpidem			
Mean daily dose	-	-	10 mg qd	10 mg qd
Concomitant drugs for venlafaxine XR				
Proportion taking anxiolytics	100%	100%	100%	100%
Most prescribed	Alprazolam			
Mean daily dose	0.5 mg qd	0.5 mg qd	0.5 mg tid	1 mg tid
Concomitant drugs for pregabalin				
Proportion taking antidepressants	10%	25%	50%	50%
Most prescribed	Paroxetine			
Mean daily dose	10 mg qd	10 mg qd	20 mg qd	20 mg qd

Source: Expert panel.

Blood analyses include glucose, gamma glutamyl transferase, creatinine, haemogram, sedimentation velocity, aspartate and alanine aminotransferase, urea and urinalysis.

**Table 5:** Unit costs (€)

**Resource**	**Unit cost**
Doctor visits	
General practitioner	35.05
Psychiatrist	54.01
Other specialist	42.66
Psychologist visits	21.96
Emergency attendances	108
Exams and analysis	
Blood analyses	19.35
Electrocardiogram	7.5
Thyroid function	8.5
Inpatient stays	1,061.54
Drugs	
Quetiapine 100 mg	1.04
Zolpidem 10 mg	0.18
Alprazolam 0,5 mg	0.08
Alprazolam 1 mg	0.12
Paroxetine 10 mg	0.20
Paroxetine 20 mg	0.41
Venlafaxine XR 102 mg	0.72
Pregabalin 348 mg	2.80

Source: [20-23].

## Results

### Base case scenario

Estimated results of both clinical outcomes and economic consequences were as expected, i.e., pregabalin is associated with better clinical results but also with higher costs. It should then be ascertained if the extra benefits compensate the extra expense. The mean HAM-A score at model entry was 27.17. For those patients taking pregabalin this score was decreased to 10.65 at week 8 and maintained through the rest of the year, while for those on venlafaxine XR the HAM-A score reduced just to 12.80. Moreover, pregabalin also allowed patients to spend more time in a better health state: during one year, the number of weeks with no or minimal GAD was estimated to be 12.9 for pregabalin and 3.8 for venlafaxine XR. Concerning the primary measure of this analysis, quality adjusted life years, pregabalin was associated to 0.738 while venlafaxine XR patients only achieved 0.712, implying a gain of 0.026. It should be stressed that, in the base case scenario, these gains were estimated assuming no dropouts and, consequently, the efficacy rates estimated for those patients that completed the clinical trial.

Again, these gains are achieved at the expense of higher costs. In fact, considering only drug costs of the comparators under assessment, pregabalin costs 780€ more during one year (1,040€ for pregabalin vs. 260€ for venlafaxine XR). However, if other healthcare resources are included, given the best prognosis of patients taking pregabalin and consequent lower resource consumption, the incremental cost is just 715€ (1,973€ for pregabalin vs. 1,258€ for venlafaxine XR). Therefore the incremental cost per QALY is 27,199€, and the incremental cost per week with no or minimal symptoms is 79€. Even if savings due to better prognosis were not included, as they rely on the resource consumption pattern estimated through the expert panel, the incremental cost-effectiveness ratios would be 29,400€ and 85€.

### Sensitivity analysis

One-way deterministic sensitivity analysis was performed on the time horizon, assuming 8 weeks and 24 weeks; on the utility values considered, evaluating the impact of using the ones obtained on the PEACE study (0.83 for HAM-A score ≤ 9; 0.71 for HAM-A 10–15; 0.61 for HAM-A 16–24; and 0.36 for HAM-A ≥25); on costs, by assuming that they could be sub or over estimated in 20%; and on the assumption regarding the inexistence of quitters. For this last sensitivity analysis it was assumed that patients could discontinue therapy due to adverse events or lack of efficacy and that, according to the expert panel, they would switch to paroxetine, whose mean change from baseline was set at 50%, based on published data [24-27]. This was also assumed for those patients for whom the panel indicated a concomitant use of venlafaxine and pregabalin, in which the switch specified by the panel was to alprazolam, i.e., in this sensitivity analysis it was assumed no difference in costs and efficacy between alprazolam and paroxetine (despite the fact that the latter is more expensive and more efficacious).

The analysis presented on Table 6 shows that results are robust, i.e., are not influenced by the hypotheses assumed. Only if the time horizon was set to 8 weeks, there would be an important increase on the incremental cost utility ratio. In fact, for a time horizon of 8 weeks the cost per QALY is 58,093€. However, it should be clarified that the time horizon for economic evaluations of drugs is often extended beyond the duration of the clinical trial. This is so because clinical trials are often too short to evaluate the onset of therapeutic failure (being the main exception those carried out on cancer drugs). Therefore, it is usually assumed that, to some extent, the clinical gains remain after the period correspondent to the clinical trial. In this study, we assumed that the difference between therapies would be maintained, while in reality it may shorten or enlarge.

**Table 6:** One-way sensitivity analysis

**Parameter**	**Cost per QALY (€)**
Time horizon	
8 weeks	58,093
24 weeks	29,580
Discontinuation and switch to paroxetine	26,860
Utilities from PEACE study [9]	21,534
Health care costs	
- 20%	27,712
+ 20%	26,868

For all other parameters, the impact of the assumptions used in the base case was modest, being important to highlight the non significant increase in the cost per QALY when discontinuation due to lack of efficacy and adverse events (and consequent switch) was assumed.

**Figure 2:**
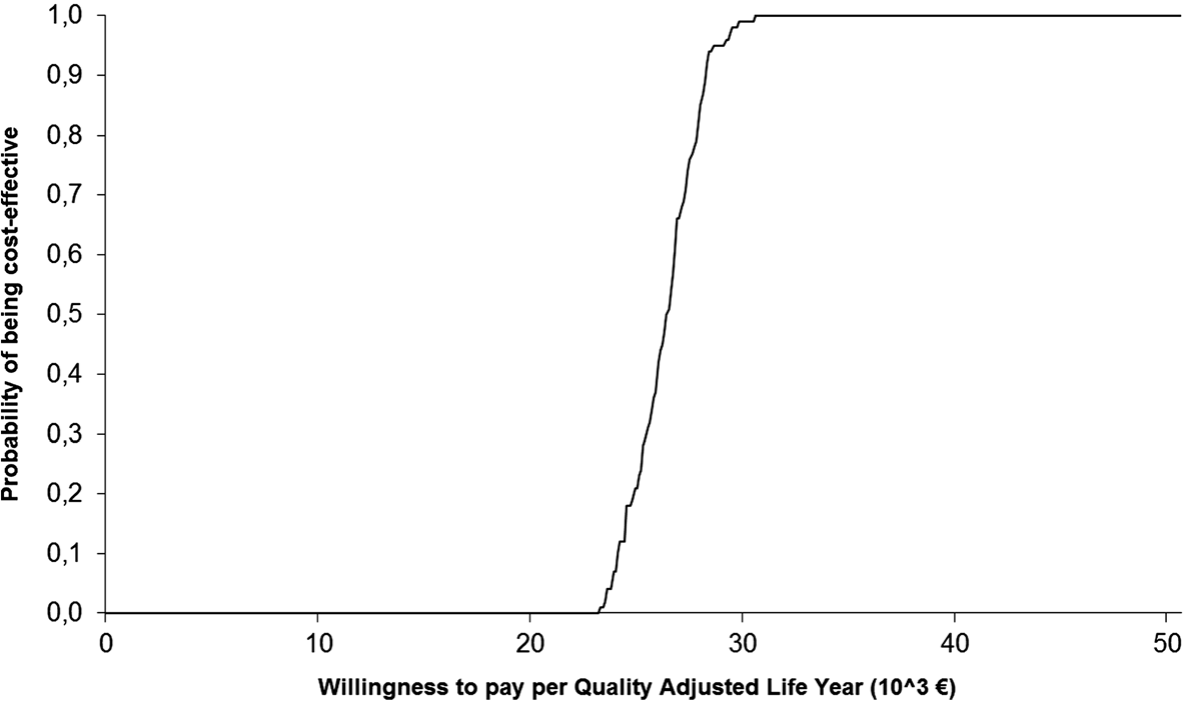
Cost-effectiveness acceptability curve.

The cost-effectiveness acceptability curve derived from the probabilistic sensitivity analysis on the weekly mean percentage changes from baseline in the HAM-A score is shown on Figure 2. It is possible to see that 90% of the cases are below an ICER of 28.2 thousand euros.

## Discussion and conclusion

In this pharmacoeconomic analysis we compared the use of pregabalin and venlafaxine XR in the treatment of generalized anxiety disorder in Portugal. The clinical side of this economic evaluation relies on a single clinical trial, in which a direct comparison between the alternatives considered was performed. In most cases, there are no direct comparisons between active treatments, so while the reliance on a single clinical study could be a weakness of this analysis, the strength derived from the direct comparison should also be acknowledged. Furthermore, the trial considered is the only one comparing flexible doses of pregabalin and venlafaxine XR, which is a more appropriate approach than assuming fixed doses. The placebo-adjusted effect size of pregabalin in this trial is similar to the effect estimated in other trials [28-32].

Concerning economic inputs, this analysis do not rely on collected data but rather on the consensus elicited via an expert panel. This is a common feature on Portuguese economic evaluations that evaluate treatments provided on an ambulatory setting, as there are no national databases of resource consumption. However, both the sensitivity analysis on costs and the small difference between the incremental cost-utility ratios with or without the cost savings due to the better health states of those patients taking pregabalin show that the expert panel findings do not influence the results. It should also be stressed that we assumed the mean dose used during the clinical trial, while it could be argued that after the first 8 weeks of modelling the mean final dose should be used. However, this was a conservative assumption as the incremental cost of pregabalin compared to venlafaxine XR is higher for the mean doses than for the final doses.

The assumption of no discontinuation could also be discussed, as it may be unrealistic, but it makes it possible to differentiate the consequences attributable to the initial treatments from the options that patients could change to. The sensitivity analysis also showed that this assumption does not impact the results significantly. Moreover, if those who discontinued treatment were included assuming that they had consumed one of the comparators without achieving any clinical gain, the ICER would decline below 23,000€.

Waxing and waning effects are not explicitly included in the model. This limitation implies that real absolute effectiveness of both drugs may differ from the estimated effectiveness. However, it should not impact incremental results, as those effects should impact results of both drugs to the same extent.

An aspect that should also be discussed is the perspective under which this analysis was conducted. In fact, we assumed a payers perspective, implying that the full cost of all resources must be included. However, as pregabalin is an anticonvulsant agent, patients only pay 10% of its price, while the nominal copayment rate for venlafaxine XR is 63%. So, under the patients perspective, pregabalin is a dominant option, i.e., it is both cheaper and more efficacious.

To our knowledge, there are only three economic studies comparing pregabalin to venlafaxine XR [8,9,33]. NICE [8] performed an economic evaluation based on a network meta-analysis in which the probability of discontinuation due to serious adverse events and the probability of response in patients without those adverse events were estimated. Pregabalin was associated with less adverse events (8.6% vs. 14.2%) and a lower conditional response probability (59.0% vs. 61.6%). Therefore, concerning response, the results of this network meta-analysis are in conflict with the conclusions of the only clinical trial directly comparing flexible doses of both drugs [16].

Either in NICE evaluation or in the first application of the model used in this study [9] it is concluded that pregabalin is associated to more QALYs. However, given the divergences in clinical inputs, the magnitude of the gains is different.

In a recent work [33], the authors also concluded for the cost-effectiveness of pregabalin vs. SSRIs/SNRIs in benzodiazepine-refractory outpatients with GAD.

In this study, assuming a threshold of 30,000€ per QALY, it is concluded that pregabalin is cost-effective in comparison with venlafaxine XR in the treatment of patients with generalized anxiety disorder in the Portuguese context.

## Competing interests

This economic evaluation was fully financed by Pfizer. Luis Silva Miguel is a full time employee of CISEP which was a paid consultant to Pfizer Portugal for the development of the economic evaluations and for the development of the manuscript. Mónica Inês is a Pfizer employee.

## Authors’ contributions

LSM and MI adapted the model to the Portuguese clinical setting and executed the economic analysis. All authors interpreted the study results. LSM and NSM carried out the expert panel elicitation. All authors were involved in the writing, review and approval of this paper. Data in this paper were previously presented as a poster at the 12nd National Conference of Health Economics held in Lisbon (Portugal), 2011.
